# 

**Published:** 1821-02

**Authors:** 


THE LONDON

### Medical and Physical Journal.

2 OF VOL. XLV.] FEBRUARY, 1821. [NO. 264.

### NOTICE.

*Another of the series of* Pröemia *to the several volumes of this Journal, (which commenced with that to the forty-third volume,) comprising a History of the Progress of Medicine and its auxiliary Sciences for the half-year immediately previous to the period of their production, respectively, was published on the last day of January. One of the especial intentions of those* Pröemia, *is to present a comprehensive view of the state and progress of Medicine throughout Europe generally, and in the United States of America; an object that cannot be effected in the regular monthly Numbers of the Journal, because of the small extent of space which can be there appropriated to this purpose.*

### MONTHLY CATALOGUE OF BOOKS.

General Elements of Pathology. By Whitlock Nicholl, M.D. 8vo.

Practical Electricity and Galvanism; containing a series of Experiments, calculated for the use of those who are desirous of becoming acquainted with that branch of Science. Second Edition. By J. Cuthbertson. 8vo.

An Inquiry into the Nature and Treatment of Gravel, Calculus, and other Diseases connected with a deranged Operation of the Urinary Organs. By Robert Prout, M.D. 8vo. 7s. 6d. Boards.

Arnott's Cases, illustrating the improved Treatment of Stricture in the Urethra and Rectum by the new Instrument, the Dilator. 8vo. 4s. 6d. boards.

Practical Observations in Midwifery, with a Selection of Cases. Part I. By John Ramsbotham, M.D. 8vo. 10s. 6d.

Practical Observations on Chronic Affections of the Digestive Organs, and on Bilious and Nervous Disorders. By John Thomas, M.D. 8vo. 6s.

Thomson's System of Chemistry. Sixth Edition. 4 vols. 8vo. 3l.

Dictionary of Chemistry. By Andrew Ure, M.D. One large 8vo. vol. 1l. 1s.

Cooke's History of the various Species of Palsy; with the Method of Cure. Being the First Part of the Second Volume of Dr. Cooke's Treatise on Nervous Diseases. 8vo. 6s. boards.

[HIGHLEY AND SON, FLEET-STREET.]

---

### TO CORRESPONDENTS.

*Several Communications were received about the commencement of the last month, that, from their relation to others which had appeared in the Journal, or their peculiar nature, should have been inserted with as little delay as possible. Our apology for their not appearing in this Number is, that (in consequence of the Proëmium occurring in the past month) it was printed off unusually early. These remarks apply especially to the Papers of* Dr. GRATTAN *and* Mr. WALLIS.

*Notwithstanding the great extent to which the Journal has, of late, been occupied by Original Communications, we view with much pleasure the large packet of them which is still before us; for, though it has become necessary that we should select from them with much restriction, a very great proportion of them is of so valuable a kind, that we cannot but be gratified on the reception of them. The writers will, we are confident, readily excuse the necessary delay in the publication of them; for, the necessity of this delay, under the circumstances above alluded to, must be as pleasing to the Readers of the Journal as it is to the Editor.*

*We offer our best thanks to the Oxford Correspondent who has of late shown so much assiduity in our favour; but we cannot insert the account given in his last letter without giving at the same time some authority for the facts related in it. None of our Readers, we are assured, would think statements of assumed facts, speaking with a general allusion, worthy of consideration, unless they are in some way or other authenticated.*

---

### TO SUBSCRIBERS.

*The increased value of the back Numbers of this Journal,* since the publication of the GENERAL INDEX to the FORTY VOLUMES, *having caused so considerable a demand, as to oblige the Proprietors to reprint several of the early Numbers, they have determined upon a* TEMPORARY REDUCTION *in the* PRICE *of all the* NUMBERS *comprised in the* FORTY VOLUMES, *(being No.* 1 *to* 238;) *which will, therefore, be sold,* during the next six months, *at* 1*s.* 6*d. each: after which time, they can only be had at the usual price of* 2*s.* 6*d.*

*** THE GENERAL INDEX *to the London Medical and Physical Journal, from Volume* 1 *to* 40 *inclusive, comprising an Analytical Table of their Contents, arranged in Alphabetical Order, with reference to the whole of the cited authorities under their Nominal Characters, &c. was lately published by* JOHN SOUTER, 73, *St. Paul's Church-yard.*

*Persons finding any difficulty in procuring the progressive* NUMBERS *of the* JOURNAL, *the* PROËMIA, *or the* GENERAL INDEX, *will please to address a line to the Publisher, as above, which will ensure a removal of every difficulty.*

